# *In Vivo* Safety and Persistence of Endoribonuclease Gene-Transduced CD4+ T Cells in Cynomolgus Macaques for HIV-1 Gene Therapy Model

**DOI:** 10.1371/journal.pone.0023585

**Published:** 2011-08-17

**Authors:** Hideto Chono, Naoki Saito, Hiroshi Tsuda, Hiroaki Shibata, Naohide Ageyama, Keiji Terao, Yasuhiro Yasutomi, Junichi Mineno, Ikunoshin Kato

**Affiliations:** 1 Center for Cell and Gene Therapy, Takara Bio Inc, Otsu, Shiga, Japan; 2 Tsukuba Primate Research Center, National Institute of Biomedical Innovation, Tsukuba, Ibaraki, Japan; Beckman Research Institute of the City of Hope, United States of America

## Abstract

**Background:**

MazF is an endoribonuclease encoded by *Escherichia coli* that specifically cleaves the ACA sequence of mRNA. In our previous report, conditional expression of MazF in the HIV-1 LTR rendered CD4+ T lymphocytes resistant to HIV-1 replication. In this study, we examined the *in vivo* safety and persistence of MazF-transduced cynomolgus macaque CD4+ T cells infused into autologous monkeys.

**Methodology/Principal Findings:**

The *in vivo* persistence of the gene-modified CD4+ T cells in the peripheral blood was monitored for more than half a year using quantitative real-time PCR and flow cytometry, followed by experimental autopsy in order to examine the safety and distribution pattern of the infused cells in several organs. Although the levels of the MazF-transduced CD4+ T cells gradually decreased in the peripheral blood, they were clearly detected throughout the experimental period. Moreover, the infused cells were detected in the distal lymphoid tissues, such as several lymph nodes and the spleen. Histopathological analyses of tissues revealed that there were no lesions related to the infused gene modified cells. Antibodies against MazF were not detected. These data suggest the safety and the low immunogenicity of MazF-transduced CD4+ T cells. Finally, gene modified cells harvested from the monkey more than half a year post-infusion suppressed the replication of SHIV 89.6P.

**Conclusions/Significance:**

The long-term persistence, safety and continuous HIV replication resistance of the *mazF* gene-modified CD4+ T cells in the non-human primate model suggests that autologous transplantation of *mazF* gene-modified cells is an attractive strategy for HIV gene therapy.

## Introduction

Highly active anti-retroviral therapy (HAART) is widely used for human immunodeficiency virus (HIV) therapy and involves the combination of several drugs with different functions that are currently being evaluated in clinical trials; some of these drugs are currently available [1]. HAART treatment reduces plasma viral load to undetectable levels and recovers CD4+ T cells to clinically safe levels. Although HAART therapy has revolutionized the treatment of HIV-1 infection, the need for life-long therapy, difficulties with medication adherence and long-term medication toxicities have led to the search for new treatment strategies that will efficiently reduce the viral load and allow for stable immunological homeostasis. The number of patients who are HAART resistant has significantly decreased in the past 2 years due to newly available drugs, but based on previous experience, drug resistance is likely to increase again. Thus, additional approaches for the management of HIV infection, or approaches performed in combination with HAART therapy, are needed. Gene therapy for HIV-1 infection has been proposed as an alternative to antiretroviral drug regimens [2], [3]. A number of different genetic vectors with antiviral payloads have been utilized to combat HIV-1, including antisense RNA against the HIV-1 envelope gene, transdominant protein RevM10, ribozymes, RNA decoys, single chain antibodies, and RNA-interference [4], [5]. These protocols use T cells or hematopoietic stem cells as a target for gene modification. Autologous T cell transfer in HIV patients began in the mid 1990's, and since that time, no serious adverse events have been reported to be associated with infusions of autologous T cells, and infusions are well tolerated. The majority of these clinical trials used gene transfer by retrovirus or lentiviral vectors for the delivery of the anti-HIV payloads.

In order to develop a new approach for HIV therapy, we previously constructed an HIV-1 Tat-dependent expression retroviral vector in which the *Escherichia coli* (*E. coli*) endoribonuclease gene *mazF* was fused downstream of the trans-activation response element (TAR) so that the gene expression of *mazF* is induced upon HIV-1 replication [6]. When MazF-transduced cells were infected with HIV-1 IIIB, the replication of HIV-1 was efficiently inhibited without affecting CD4+ T cell growth. MazF-transduced primary CD4+ T cells derived from monkeys also suppressed simian/human immunodeficiency virus (SHIV) replication [6]. Thus, autologous transfer of genetically modified CD4+ T cells conditionally expressing the MazF protein will be a promising strategy for HIV gene therapy. Generally, the shift from the chronic phase to the AIDS phase is due to the balance between viral growth and immune suppression, and the remarkable decrease in CD4+ T cells causes the subsequent deficiency of the immune system, the hallmarks of AIDS. The benefit of the MazF-based gene therapy strategy is that gene-modified CD4+ T cells may be protected from HIV-1-associated cell death and are therefore likely to help the immune system maintain a stable condition.

In this preclinical study, we examined the *in vivo* safety and persistence of MazF-transduced autologous CD4+ T cells (named MazF-Tmac cells) using a non-human primate model. Cynomolgus macaque primary CD4+ T cells were retrovirally transduced with the MazF vector, infused into the autologous monkeys, and the persistence and safety of the MazF-Tmac cells was monitored more than half a year. We found that infused MazF-Tmac cells were detected in the peripheral blood throughout the experimental period. Additionally, experimental autopsy revealed the distribution of the infused lymphocyte in total body.

## Results

### Manufacturing of MazF-transduced CD4+ T cells using *ex vivo*-expanded cynomolgus macaque CD4+ T cells

**Figure 1:**
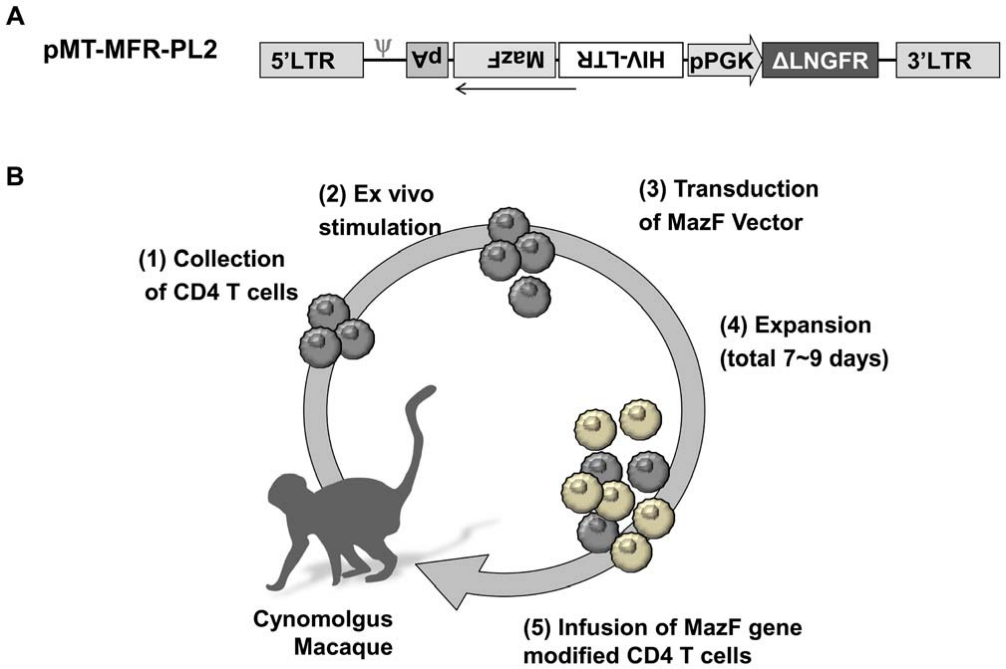
Diagram of autologous CD4+ T cell transplantation using a non-human primate model. (A) Design of gene transfer vector. The MazF gene derived from *E. coli* was inserted directly into the downstream of HIV-LTR sequence. The HIV-LTR-MazF-polyA cassette was introduced in the opposite direction of the MoMLV-LTR. A truncated form of the human ΔLNGFR was also introduced into the retrovirus vector as a surface marker. The ΔLNGFR gene is under the control of the human PGK promoter. (B) Flow diagram of gene-transduced CD4+ T cell manufacture. (1) Peripheral blood was collected by apheresis, (2) CD4+ T cells were selected by positive selection and stimulated *ex vivo* with Con A or anti-CD3/CD28 monoclonal antibody-conjugated beads. (3) The MT-MFR-PL2 vector was transduced twice on days 3 and 4. (4) The transduced cells were expanded for an additional 3–5 days until the total cell number reached more than 10^9^. (5) On day 7–9, the expanded cells were collected, washed, and infused to the autologous macaques through venous blood.

In order to infuse more than 1×10^9^ MazF-transduced autologous cells, isolated primary CD4+ T lymphocytes were *ex vivo* stimulated, transduced with the MT-MFR-PL2 retroviral vector (Figure 1A), and expanded as described in the Materials and Methods. The resultant MazF-Tmac cells were transplanted into autologous monkeys via intravenous infusion (Figure 1B). We initially used concanavalin A (Con A) for the stimulation of CD4+ T cells (CD4T-1), but Con A only induced a 12-fold cell expansion after 7 days. In order to improve the *ex vivo* expansion, we used anti-CD3/anti-CD28 monoclonal antibody-conjugated beads (anti-CD3/CD28 beads), which are known to yield a more efficient cellular expansion [7], [8]. As we expected, the fold expansion of CD4+ T cells (CD4T-2 and CD4T-3) stimulated with anti-CD3/CD28 beads was much higher than with Con A stimulation (Table 1). In order to improve the engraftment efficiency of CD4+ T cells, busulfan was orally administered to the macaques prior to the transplantation, and the gene-modified MazF-Tmac cells were infused into each monkey intravenously at 1.6–2.7×10^9^ cells.

**Table 1:** Demographic data and summary of expansion fold and transduction efficiency.

	CD4T-1	CD4T-2	CD4T-3
Body Weight (kg)	5.25	5.18	3.7
Method for stimulation	Con A	Anti-CD3/CD28 Beads	Anti-CD3/CD28 Beads
Number of stimulated CD4+ T cells (×10^7^ cells)	13.0	1.0	4.6
Days for expansion (days)	7	7	9
Number of infused MazF-Tmac cells (×10^9^ cells)	1.6	1.7	2.7
Expansion Fold	12.3	170	58.7
Gene transfer efficiency (%)	34.5	61.8	60.0

### Transduction efficiency and cell surface markers of MazF-Tmac cells

The efficiency of MazF transduction and phenotype of cell surface markers of the MazF-Tmac cells were analyzed using flow cytometry. The MazF vector transduction efficiency of CD4T-2 and CD4T-3 cells was 61.8% and 60.0%, respectively, while only 34.5% for CD4T-1 (Table 1). As shown in Table 2, 99% of the expanded MazF-Tmac cells were CD3 and CD4 double-positive, and in these cells, more than 90% expressed CD95/CD28, which are known central memory phenotype markers [9]. Central memory cells generally have a longer life span compared to effecter memory cells [10]; thus, a higher percentage of central memory cells in MazF-Tmac cells is likely to result in longer persistence after transplantation. Furthermore, to assess the activation status of MazF-Tmac cells, we measured the expression of CD25, which is also known as IL-2 receptor alpha and is an activated T cell marker. CD25 expression of MazF-Tmac cells from CD4T-2 and CD4T-3 was low. In contrast, almost 100% of the CD4+ T cells were found to express CD25 with a higher expression level 2–4 days after stimulation (data not shown). Thus, these data indicate that a large number of MazF-Tmac cells entered into resting or non-activated states during the *ex vivo* culture. CXCR4, a co-receptor for X4 tropic HIV entry, was found to be expressed in expanded CD4T-2 and CD4T-3 MazF-Tmac cells. Furthermore, we observed that there was no significant difference in the measured cell surface markers between Con A- and anti-CD3/CD28 bead-stimulated MazF-Tmac cells (Table 2).

**Table 2:** Cell surface markers of expanded MazF-Tmac cells.

	CD4T-1	CD4T-2	CD4T-3
CD3(+)/CD4(+) (%)	98.2	98.7	99.9
CD95(−)/CD28(+) (Naïve) (%)	0.7	1.2	0.4
CD95(+)/CD28(+) (CM) (%)	93.0	94.7	91.2
CD95(+)/CD28(−) (EM) (%)	6.2	3.9	8.3
CXCR4 (%)	N/A	92.0	79.4
CD25 (%)	N/A	30.4	24.5

CM: Central Memory, EM: Effector Memory.

### Longitudinal analysis of infused MazF-Tmac cells

**Figure 2:**
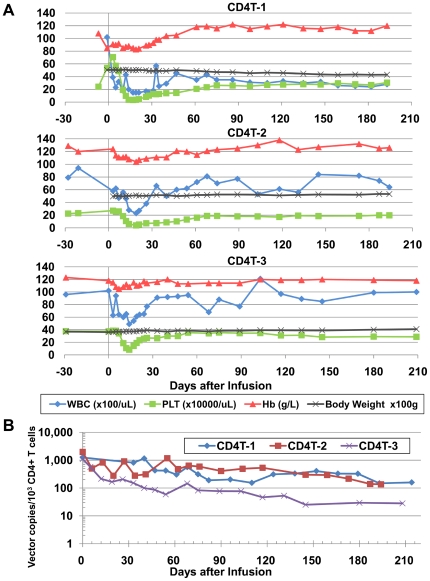
Hematological analysis and engraftment of the MazF-transduced CD4+ T cells. (A) The body weight and several hematological features were measured at the indicated time points, and the number of WBC, Hb, and PLT were represented. Each macaque was monitored throughout the study period. (B) The *in vivo* persistence of retroviral-transduced CD4+ T cells in the peripheral blood. PBMCs were collected at the indicated time points. The percentage of CD4+ T cells was analyzed using flow cytometry, and the proviral MazF vector copy was analyzed using real-time PCR. By compounding these two data, the copy number of the *mazF* gene in CD4+ T cells was calculated.

To examine the *in vivo* safety and persistence of infused MazF-Tmac cells, peripheral blood from each monkey was collected to monitor the hematological effects and the proviral copy number of the transduced retroviral vector in the genome over six months. There was no significant change in the body weight of the monkeys throughout the experiment (Figure 2A). During the period of 2–4 weeks post-transplantation, severe reduction in the white blood cell (WBC) count, hemoglobin (Hb) concentration, and platelet (PLT) levels were observed in the monkeys CD4T-1 and CD4T-2, while only slight reduction was observed in CD4T-3. These negative effects are considered to be due to the effect of the busulfan treatment, which is known to cause partial bone marrow depletion and functional defects in blood-forming tissues. No other adverse events were observed throughout the experiments. The transient reduction of lymphocytes gradually recovered, and the cell number became stable two months after the transplantation (Figure 2A).

The percentage of persistent MazF-Tmac cells in CD4+ T cells was determined using real-time PCR and flow cytometric analyses. The percentage of MazF-Tmac cells gradually decreased in CD4T-1- and CD4T-2-transplanted monkeys, while in the CD4T-3-transplanted monkey, a drastic reduction of the infused MazF-Tmac cells was observed 3–4 weeks post-transplantation but was not observed at later time points (Figure 2B). Although the levels of MazF-Tmac cells gradually decreased over time, the infused MazF-Tmac cells were detected even after six months post-transplantation. It is reasonable to assume that a population of infused MazF-Tmac cells can persist for a long-term period, likely forming a resting condition.

### Detection of anti-MazF antibodies in monkey blood

**Figure 3:**
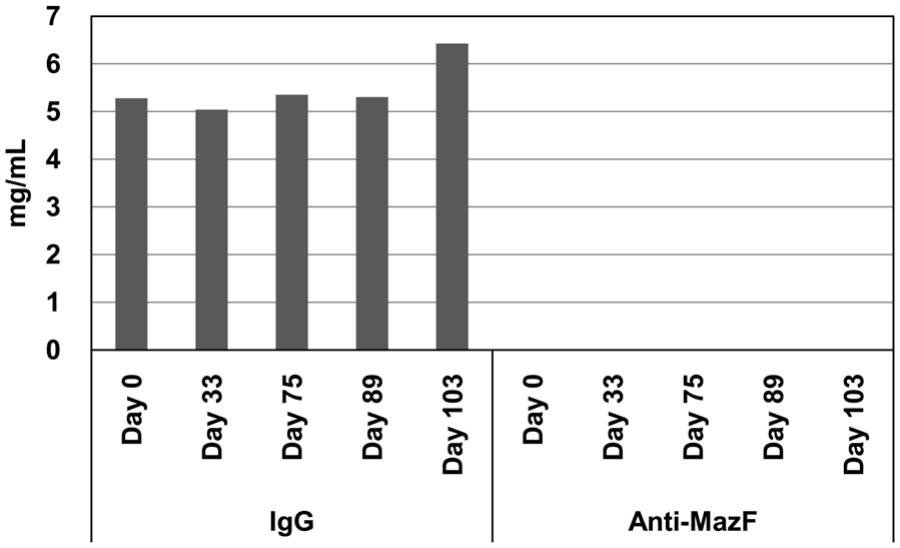
No detection of anti-MazF antibodies in monkey blood after transplantation of MazF-Tmac cells. Plasma samples were isolated from the monkey CD4T-2 at day 0, 33, 75, and 103 after transplantation and were used to detect anti-MazF antibodies on a MazF protein-immobilized microplate. The plasma samples were diluted to 500,000-fold, 50,000-fold, and 10,000-fold and added to each well. After the incubation, antibodies which reacted with immobilized MazF were tried to detect as described in Materials and Methods. No MazF-specific antibodies were detected.

Although the levels of MazF-transduced CD4+ T cells gradually decreased in the peripheral blood, some were detected throughout the half-year experimental period, suggesting that MazF-Tmac cells showed little or no immunogenicity towards cynomolgus macaques. Because gene therapy for HIV is aimed at reconstituting an HIV-resistant immune system, genetically modified cells must not only inhibit virus replication, but also maintain their expected trafficking behavior and persist *in vivo*. Although the evidence of longitudinal persistence of MazF-Tmac cells supports the low immunogenicity of MazF-Tmac cells, it is important to assess the production of antibodies against MazF. As shown in Figure 3 and Figure S1, we detected no production of anti-MazF antibodies in the CD4T-2 monkey blood after transplantation of the MazF-Tmac cells.

### *In vivo* safety of MazF-Tmac cells

It is a great advantage to use primate models for investigating the safety of gene-modified cells, as they can be used for surgical pathological analysis. Therefore, we performed experimental autopsies six months after transplantation. To examine the safety of MazF-Tmac cells, specimens from several organs were fixed in buffered formaldehyde and embedded in plastic. Serial sections were made using a diamond saw. Slides were then stained with hematoxylin-eosin. Histopathological findings of the specimens were contracted with Bozo Research Center (Tokyo, Japan), and no severe adverse events relating to MazF-Tmac cell infusion was observed (Table 3 and Figure S2).

**Table 3:** Analysis of *in vivo* safety (Histological finding about autopsy sample).

	CD4T-1	CD4T-2	CD4T-3
Lymph node	±	±	−
Spleen	−	−	±
Bone marrow	++**	−	−
Thymus	N/A	+*	−
Small intestine	−	−	−
Liver	−	−	−
Kidney	−	±	−
Pancreas	−	−	−
Stomach	−	−	±
Lung	−	±	−
Heart	−	−	±

−: No remarkable changes; ±: Minimal; +: Mild; ++: Moderate.

N/A: No equivalent sample available.

* Due to the Aging,

** Side effect due to the busulfan administration.

### Examination of the anti-viral efficacy of MazF-Tmac cells harvested from monkey

**Figure 4:**
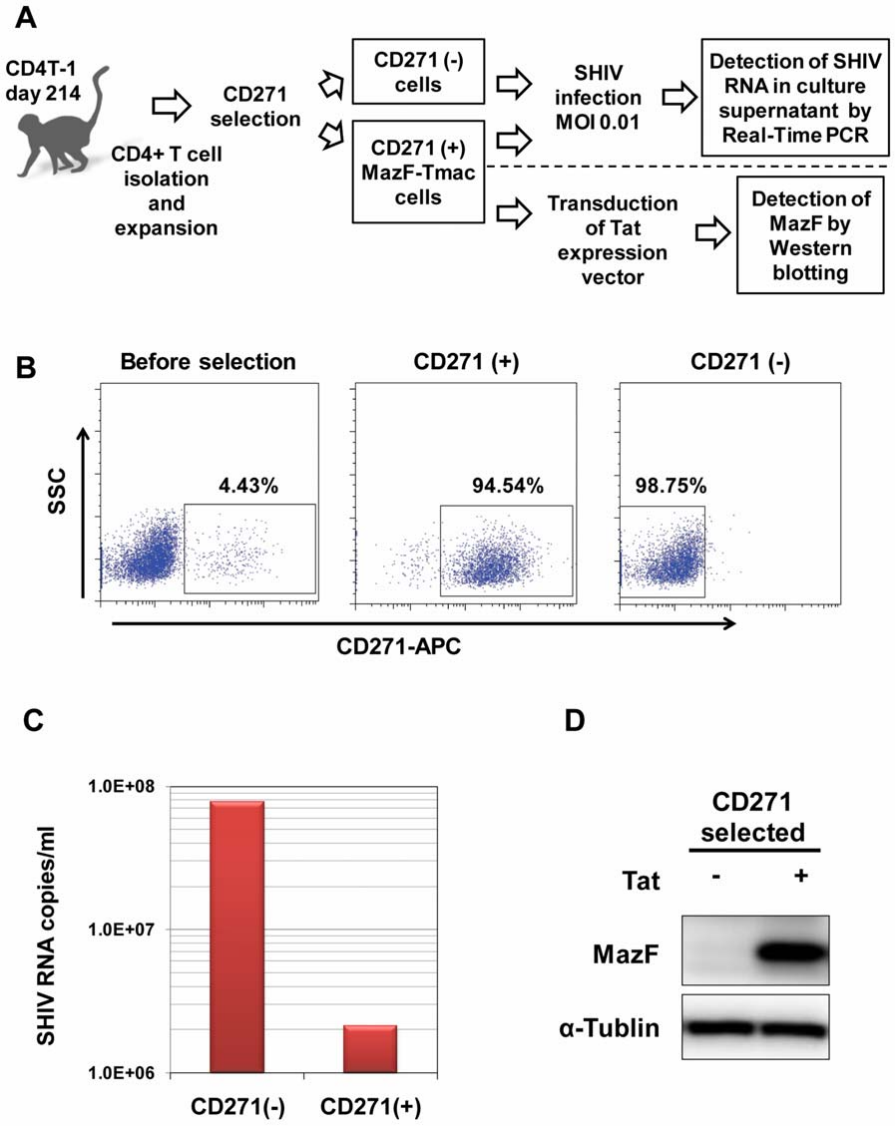
Examination of the anti-viral efficacy of MazF-Tmac cells harvested from the monkey. (A) Flow diagram of the experiment. CD4+ T lymphoid cells from CD4T-1 (214 days post-infusion of the MazF-Tmac cells) were stimulated and expanded *ex vivo*. The genetically modified cells expressing ΔLNGFR+ were concentrated with an anti-CD271 monoclonal antibody and expanded for 4 days. The expanded CD271-enriched cells and CD271-negative cells were infected with SHIV 89.6P. SHIV RNA levels in the culture supernatant were determined using quantitative real-time PCR. Expression of MazF was detected from the cell lysates by western blot analysis. Moreover, CD271-positive cells were transduced with the Tat expression vector. (B) CD271-positive and -negative cells were enriched using an anti-CD271 antibody, and dot plots of the flow cytometry analysis are presented. (C) The suppression of SHIV RNA in the culture supernatant at 6 days after infection was detected by real-time PCR analysis. (D) MazF-Tmac cells transduced with the Tat expression vector were harvested at 20 hours post-transduction and used for western blot analysis. Conditional expression of MazF in a Tat-dependent manner was observed.

In order to examine whether the Tat-dependent expression of MazF and anti-viral efficacy was maintained in the MazF-Tmac cells after infusion, CD4+ T lymphoid cells from a CD4T-1-transplanted monkey (214 days post-infusion of MazF-Tmac cells) were selected and expanded *ex vivo* (Figure 4A). After 7 days of expansion, the genetically modified cells expressing a truncated form of the human low affinity nerve growth factor (ΔLNGFR+) were concentrated with an anti-CD271 monoclonal antibody (Figure 4B). CD271-positive cells and CD271-negative cells were expanded for an additional 4 days. Both groups of expanded cells were infected with SHIV 89.6P [11] at the multiplicity of infection (MOI) of 0.01. Culture supernatants and cell pellets were analyzed at 6 days post-infection. As shown in Figure 4C, the replication of SHIV 89.6P was significantly suppressed in CD271-positive cells in comparison with CD271-negative cells. Although western blot analysis managed to detect the expression of MazF, MazF was below the detection limit (data not shown). However, the expression of MazF was clearly induced when the same CD271-positive cells were transduced with the Tat expression retroviral vector M-LTR-Tat-ZG [6] (Figure 4D). These data suggest that the conditional expression system in MazF-Tmac cells is still active at 6 months post-transplantation.

### Distribution of MazF-Tmac cells

**Figure 5:**
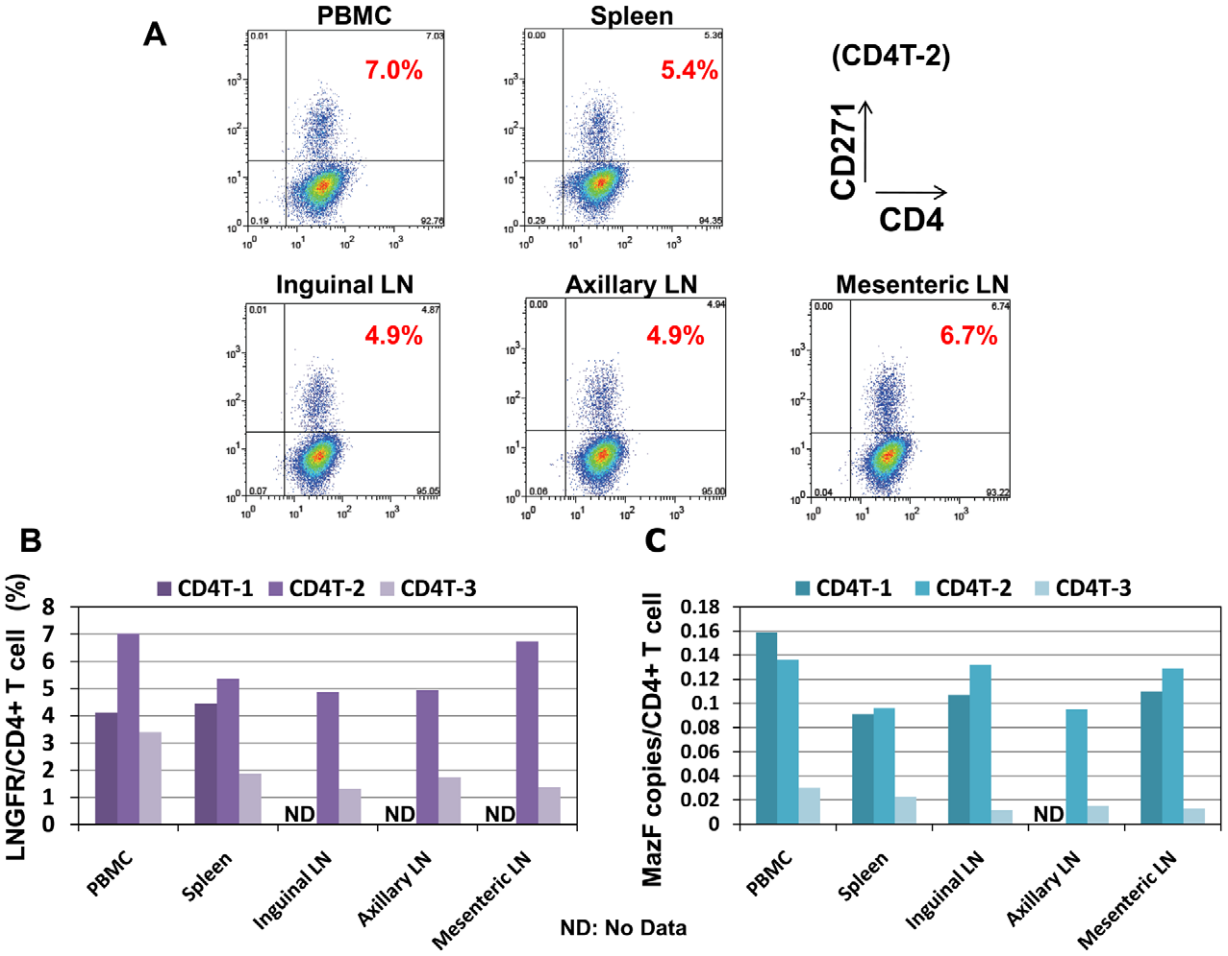
Analysis of the distribution of MazF-Tmac cells in several organs. (A) CD4+ T cells were isolated from lymphocytes separated from several organs, incubated 3–4 days, and stained with anti-CD4 and anti-CD271 antibodies. CD4T-2 is represented by a dot plot. (B) The percentage of CD271+ cells from three macaques is summarized. (C) The Copy number of the MazF gene in CD4+ T cells from each organ was calculated from real-time PCR and flow cytometric data.

To examine the distribution and persistence of the infused MazF-Tmac cells in a monkey, lymphocytes isolated from several organs were analyzed using flow cytometry and real-time PCR. As shown in Figure 5A and 5B, ΔLNGFR+ cells were detected in CD4+ T cells isolated from several lymph nodes (LNs), spleen, and peripheral blood. A similar tendency was obtained using real-time PCR (Figure 5C). In contrast, MazF-Tmac cells were not detected in the bone marrow, liver, thymus, and small intestine (data not shown). These data strongly suggest that infused MazF-Tmac cells mainly circulate in the secondary lymphoid organs.

### *In vivo* distribution of MazF-Tmac cells treated with or without retinoic acid

**Figure 6:**
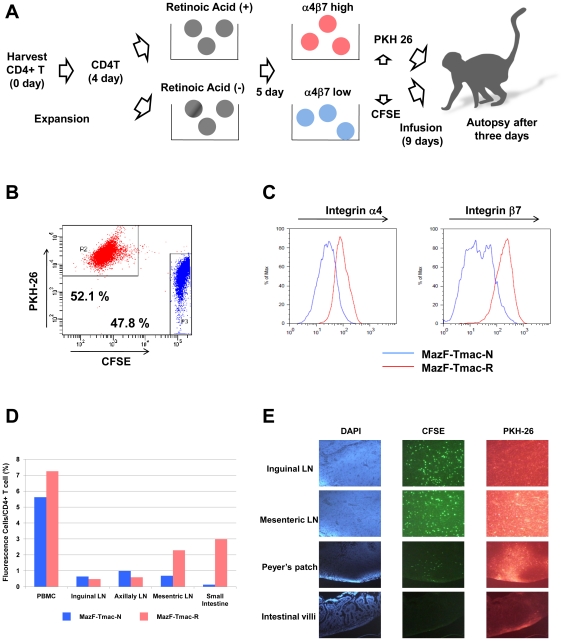
Comparison of the homing effect of MazF-Tmac cells treated with or without retinoic acid. (A) CD4+ T cells from the CD4T-4 monkey were stimulated with anti-CD3/CD28 beads, and MT-MFR-PL2 vector was transduced twice on days 3 and 4. After transduction, total lymphocytes were divided into two culture conditions in which retinoic acid was added to the one. After an additional 5 days of incubation, control and retinoic acid-treated cells were stained with CFSE and PKH26, respectively, mixed at nearly the same numbers, and infused into the autologous CD4T-4. Three days after the transplantation, experimental autopsy was performed. (B) A mixture of the two groups of MazF-Tmac cells stained with CFSE and PKH26 was analyzed using flow cytometry; the ratio of the two groups was almost same. (C) Up-regulation of the homing receptor was confirmed in the MazF-Tmac-R cells. The MazF-Tmac-N and MazF-Tmac-R cells are indicated by the blue line and red line, respectively. (D) Lymphocytes were collected from three lymph nodes (LNs) and small intestines, and a percentage of fluorescently-labeled cells were analyzed by flow cytometry. (E) Fluorescence microscope analysis of distal organ specimens.

Based on the findings that MazF-Tmac cells were well distributed among secondary lymphoid organs but not in small intestine, we performed additional experiment using one cynomolgus monkey (CD4T-4). In order to investigate the editing effect of the homing receptor to efficiently recruit the gene-modified cells to intestinal tissues in a non-human primate model, the distribution of retinoic acid-treated MazF-Tmac cells was examined in a cynomolgus macaque. The experimental procedure is described in Figure 6A. Non-treated and retinoic acid-treated MazF-Tmac cells were designated as MazF-Tmac-N and MazF-Tmac-R, respectively. Expressions of integrin-α4 and integrin-β7 were remarkably increased in the presence of retinoic acid (Figure 6B). Thereafter, MazF-Tmac-N and MazF-Tmac-R were labeled with carboxyfluorescein diacetate succinimidyl ester (CFSE) and PKH26, respectively. The CFSE-labeled cells were mixed with an equal number of PKH26-labeled cells (Figure 6C), and 6.8×10^8^ of the mixed cells were infused into a CD4T-4 monkey. Note that the transduction efficiency of the MazF vector was 65% (data not shown). Three days after the transplantation, experimental autopsy was performed to obtain samples of several organs as described in the Materials and Methods. Both the CFSE- and the PKH26-labeled CD4+ T cells were detected in the peripheral blood and several LNs by FACS analysis (Figure 6D). The percentage of the infused cells in the LNs was low compared to the peripheral blood, indicating that a large number of the infused cells did not migrate to the secondary lymphoid tissues and circulated in the peripheral blood at this time point. In the case of the inguinal and axillary LNs, the percentage of MazF-Tmac-R cells was low compared to MazF-Tmac-N cells. In contrast, a higher percentage of MazF-Tmac-R cells was observed in the mesenteric LN compared to MazF-Tmac-N cells. MazF-Tmac-N cells were evenly distributed in the three LNs analyzed, while the MazF-Tmac-R cells seemed to be preferentially distributed in the mesenteric LNs. Moreover, a large number of MazF-Tmac-R cells were distributed in the small intestine, while MazF-Tmac-N cells were not. To further evaluate the homing effect of the MazF-Tmac cells, the distribution of the labeled-MazF-Tmac cells in cryopreserved organs was analyzed using fluorescence microscopy (Figure 6E). A number of the PKH26-labeled MazF-Tmac-R cells were observed in the mesenteric LNs and in Peyer's patches. Taken together, retinoic acid-treated MazF-Tmac cells seem to be selectively recruited to mesenteric LNs and then transported to Peyer's patches. The distribution of MazF-Tmac-R cells in the intestinal villi remains to be determined.

## Discussion

MazF is a toxin encoded by the *E. coli* genome and plays a role in growth regulation under stress conditions in *E. coli* [12]. MazF can act as an endoribonuclease (RNase) that specifically cleaves cellular mRNAs at ACA sequences [13]. Therefore, MazF induction in *E. coli* virtually eliminates almost all cellular mRNAs to completely inhibit protein synthesis. However, MazF-induced cells retain full capacity for protein synthesis, as MazF-induced cells are able to produce a protein at a high level if the prerequisite mRNA is engineered to be devoid of all ACA sequences without altering its amino acid sequence [14]. This indicates that RNA components involved in protein synthesis are protected from MazF cleavage. Indeed, ribosomal RNAs (rRNAs) and transfer RNAs (tRNAs) are protected from MazF cleavage in *E. coli* [15].

RNase-based anti-HIV gene therapy is an attractive strategy to suppress HIV-1 RNA replication. In the case of MazF, there are more than 240 ACA sequences in HIV-1 RNA, suggesting that HIV has almost no chance to gain MazF-related escape mutations. This approach seems to have a substantial advantage over the other known antiviral strategies, including antiviral drug therapy, and RNA-based gene therapies, such as antisense RNA, ribozyme, and siRNA.

MazF overexpressed in mammalian cells preferentially cleaves messenger RNAs (mRNAs), but not ribosomal RNAs [16]. As HIV-1 RNA has more than 240 ACA sequences, we assumed that the viral RNA is highly susceptible to MazF, leading to inhibition of viral replication under a conditional expression system. Indeed, conditional expression of MazF with Tat suppresses replication of both HIV-1 IIIB and SHIV 89.6P without affecting cellular mRNAs, suggesting that this Tat-dependent expression system of MazF is an attractive payload for HIV gene therapy [6]. It is an intriguing phenomenon that viral RNAs are efficiently and preferentially cleaved without affecting cellular mRNAs, and we are now addressing this question. Meanwhile, MazF is a bacterial protein, and its expression is induced by Tat protein; thus, it is important to assess the safety and immunogenicity of *mazF* gene-modified cells *in vivo*. In order to determine the safety of our MazF-retrovirus system *in vivo*, we infused MazF-transduced CD4+ T cells into cynomolgus macaques. In human gene therapy trials, engraftment of 1–2% of genetically modified cells in the peripheral circulation has been observed following infusions of about 10 billion cells [17], and higher cell doses results in higher levels of engraftment [18], [19]. Infusions of lower than 5×10^9^ cells do not reliably result in measurable engraftment levels [19]. Therefore, we decided to infuse more than one billion cells into cynomolgus macaques, reflecting one-tenth of the scale of the human model. Indeed, the *mazF* gene-modified cells were detected over a six month period at a high level, and no histopathological disorders and no MazF-specific antibody production was observed during the experiment, demonstrating that MazF-Tmac cells showed little or no immunogenicity to monkeys. Moreover, MazF-Tmac cells harvested from the CD4T-1-transplanted monkey 6 months post-infusion showed resistance to the replication of SHIV 89.6P, indicating that the long-term persistent MazF-Tmac cells are functional. The expression of MazF in the SHIV-infected MazF-Tmac cells was below the limit of detection due to a low MOI such as 0.01, while in the MazF-Tmac cells transduced with the Tat expression retroviral vector M-LTR-Tat-ZG at 45% efficiency, expression of MazF was clearly induced, indicating that Tat dependent MazF expression system was maintained in the cells even 6 months after the autologous transplantation.

Because gene therapy for HIV is aimed at reconstituting an HIV-resistant immune system, genetically modified cells must inhibit virus replication and maintain persistence *in vivo*. Although *ex vivo* gene therapy targeting CD4+ T cells or CD34+ hematopoietic stem cells has been shown to promote long term persistence of infused cells in peripheral blood in human, it is difficult to obtain information about the distribution pattern of these cells in the whole human body. In order to obtain such information, the monkeys were sacrificed and lymphocytes were isolated from several organs after 6 months of monitoring. Importantly, the infused MazF-Tmac cells were detected in secondary lymphoid tissue, such as several LNs and spleen, and in peripheral blood, although individual differences between CD4T-1, -2, and -3-transplanted monkeys were observed. No histopathological disorders were observed in the organs containing MazF-Tmac cells, indicating that there were no lesions relating to MazF-Tmac cells. The distribution of MazF-Tmac cells in the lymphoid tissues of CD4T-3-transplanted monkey was lower compared to the CD4T-1 and -2-transplanted monkeys. One reason for this phenomenon is likely the lower dosage of busulfan used to treat the CD4T-3-transplated monkey. Busulfan is an alkylating agent with potent effects on hematopoietic stem cells that is commonly used for stem cell transplantation. In rhesus macaques, a low-dose of busulfan has an impact on bone marrow stem/progenitor cells with transient and mild suppression of peripheral blood counts [20]. Thus, the lower engraftment efficiency of CD4T-3 (MazF-Tmac) cells might be due to the milder busulfan treatment.

In contrast to the LNs and spleen, a limited number of cells were detected in non-lymphoid tissues such as small intestine and liver. Considering HIV-1 infection, the gastrointestinal (GI) tract, which contains the vast majority of lymphoid tissues in the total body to protect mucosal membranes from foreign antigens, is the dominant site of HIV replication rather than LNs, which were originally thought to be the main infection sites [21]. In GI tract, CD4+ T cells are dramatically decreased during the acute phase of HIV infection [21], [22], [23]. In rhesus macaques, a similar depletion was also reported during the acute phase of simian immunodeficiency virus (SIV) infection, with CD4+ memory T cells specifically targeted [24], [25]. Notably, the rate of mucosal CD4+ T cell depletion in pathogenic SIV-infected monkeys correlates with the disease progression in the rhesus macaque [26]. Indeed, recent studies provide evidence that the depletion of mucosal CD4+ T cells leads to damage of the gut mucosal layer resulting in translocation of microbial products, such as lipopolysaccharide (LPS), ultimately causing chronic and systemic immune activation, which is one of the hallmarks of HIV/SIV infection and one of the predictors of disease progression [27], [28]. Although HAART therapy is effective in controlling viral replication and recovering CD4+ T cells in the peripheral blood, restoration of CD4+ T cells is delayed in the GI tract [21], [29]. Thus, the repair of depleted CD4+ T cells using gene therapy might attenuate the breakdown of the mucosal layer and prevent mucosal immune system deficiency. To change the tissue distribution of infused CD4+ T cells, the enhancement of homing receptor expression in T lymphocyte is necessary. Integrin α4β7 is known to facilitate the migration of lymphocytes from gut-inductive sites where immune responses are first induced (Peyer's patches and mesenteric LNs) to the lamina propria [30], [31]. Expression of the homing receptor is induced by the addition of retinoic acid [32], which is produced mainly from retinol (vitamin A) by dendritic cells in the mesenteric LNs. As shown in Figure 6D and 6E, although these are preliminary data with only one monkey, editing of the homing receptors integrin-α4 and integrin-β7 by retinoic acid enhanced the recruitment of MazF-Tmac cells to the mesenteric LNs, small intestine, and Peyer's patches. These results may indicate that MazF-Tmac cells treated with retinoic acid selectively accumulate in the mesenteric LNs and then migrate into Peyer's patches. It has been reported that the HIV-1 envelope protein gp120 binds to and signals through the activated form of integrin α4β7 [33]; however, we expect that retinoic acid-treated MazF-T cells will persist in distal organs without the additional spread of HIV replication because of the HIV-1 resistance observed in the MazF-Tmac cells. Therefore, we speculate that the combination of several culture methods to edit the homing receptor will enhance the recruitment of MazF-Tmac cells to distal lymphoid organs, resulting in a more efficient therapeutic.

In summary, we showed long-term persistence, safety and continuous HIV replication resistance in the *mazF* gene-modified CD4+ T cells in a non-human primate model *in vivo*, suggesting that autologous transplantation of *mazF* gene-modified cells is an attractive strategy for HIV gene therapy.

## Materials and Methods

### Vector design and viral production

The GALV-enveloped gamma retroviral vector MT-MFR-LP2 was generated as previously described [6]. MT-MFR-PL2 expresses a truncated form of the human low affinity nerve growth factor gene (ΔLNGFR) [34] under the control of a functional PGK promoter and the MazF gene under control of the HIV-LTR promoter (Figure 1A). The ΔLNGFR is a surface marker that allows identification of transduced cells.

### Animals

Four cynomolgus macaques (*Macaca fascicularis*, 6–7 years old), CD4T-1, CD4T-2, CD4T-3, and CD4T-4, were used in this experiment and were maintained at the Tsukuba Primate Research Center for Medical Science at the National Institute of Biomedical Innovation (NIBIO, Ibaraki, Japan). The study was conducted according to the Rules for Animal Care and the Guiding Principles for Animal Experiments Using Nonhuman Primates formulated by the Primate Society of Japan [35] and in accordance with the recommendations of the Weatherall report, “The use of non-human primates in research”. The protocols for the experimental procedures were approved by the Animal Welfare and Animal Care Committee of the National Institute of Biomedical Innovation (DS18-100). All surgical and invasive clinical procedures were conducted by trained personnel under the supervision of a veterinarian in a surgical facility using aseptic techniques and comprehensive physiologic monitoring. Ketamine hydrochloride (Ketalar, 10 mg/kg; Daiichi-Sankyo, Tokyo, Japan) was used to induce anesthesia for all clinical procedures associated with the study protocol such as blood sampling, gene-modified cell administration, clinical examinations and treatment.

### *Ex vivo* expansion of CD4+ T cells, and transduction of the MazF vector

Peripheral blood from cynomolgus macaques was collected by apheresis as previously described [36]. For the dissolution of red blood lymphocytes, collected blood was treated with ACK lysing buffer (Lonza, Walkersville, MD) and was washed twice with phosphate buffered saline (PBS). Then, CD4+ T cells were isolated using anti-CD4 conjugated magnetic beads (Dynal CD4 Positive Isolation Kit, Invitrogen, Carlsbad, CA) according to the manufacturer's instructions. Isolated CD4+ T cells were cultured at 5×10^5^ cells/ml in GT-T503 (Takara Bio, Otsu, Japan) supplemented with 10% FBS (Invitrogen), 200 IU recombinant human interleukin-2 (IL-2; Chiron, Emeryville, CA), 2 mM L-glutamine (Lonza), 2.5 µg/ml Fungizone (Bristol Myers-Squibb, Woerden, The Netherlands) and activated for three days with either 5 µg/ml concanavalin A (Con A, Sigma Chemical, St. Louis, MO) for CD4T-1 or a combination of anti-CD3 clone FN-18 (Biosource, Camarillo, CA, USA) and anti-CD28 clone L293 (BD Biosciences, Franklin Lakes, NJ) monoclonal antibodies conjugated to M-450 epoxy magnetic beads (Invitrogen) at cell-to-bead ratio of 1∶1 (CD4T-2 and CD4T-3). On day 3, the activated CD4+ T cells were transduced with the MazF retroviral vector MT-MFR-PL2 in the presence of RetroNectin® (Takara Bio) according to manufacturer's instructions. Transduction was repeated on day 4. CD4+ T cells were further expanded to day 7 to 9 until the total cell number reach more than 10^9^. The closed system MazF-Tmac cell manufacturing was performed using gas permeable culture bags; Cultilife 215 (Takara Bio) and Cultilife Eva (Takara Bio) were used for CD4+ T cells expansion and Cultilife spin (Takara Bio) was used for transduction of the MazF retroviral vector.

### Transplantation of expanded CD4+ T cells

Prior to the transplantation, each macaque was treated with busulfan (Ohara Pharmaceutical, Shiga, Japan). Busulfan has been used extensively as a preparatory regimen for allogenic hematopoietic stem cell transplantation based on its toxicity to hematopoietic stem cells. Furthermore, it has been reported that in non-human primates, hematopoiesis was significantly decreased after a single, clinically well-tolerated dose of busulfan, with slow, but almost complete, recovery over the next several months [20]. The effects of busulfan on lymphocyte engraftment, however, are not well documented. Although cyclophosphamide is widely used in immune gene therapy trials in humans for lymphocyte transplantation, there is no information available for cyclophosphamides effect on T-cell transplantation in the cynomolgus macaque. It should be noted that we have chosen busulfan for our CD4+ T cell transplantation because busulfan is shown to cause a reduction in the peripheral blood count in human trial [37], we have had success in using busulfan for cynomolgus macaque bone marrow transplantation and according to internal information, busulfan causes a reduction of the peripheral blood count in cynomolgus macaques. Busulfan was orally administered to the macaques twice at 10 mg/kg each (CD4T-1 and CD4T-2) or 6 mg/kg each (CD4T-3) [38]. The expanded cells were harvested, washed three times with PBS, and re-suspended in PBS containing 10% autologous plasma. The collected cells were infused intravenously to monkeys at the speed of 1 ml per minute.

### Flow cytometry analysis

The cell surface markers of the expanded cells and peripheral blood mononuclear cells (PBMC) were analyzed using FACSCalibur (BD Bioscience) and FACSCanto (BD Bioscience), and data analysis was performed using CellQuest software (BD Bioscience), FACSDiva software (BD Bioscience) or FlowJo software (Tree Star, Inc., Ashland, OR). The following antibodies were used for staining: anti-CD3 (SP34-2, PerCP), anti-CD4 (L200, FITC), anti-CD25 (2A3, FITC), anti-CD28 (CD28.2, PE), anti-CD95 (DX2, FITC), anti-CXCR4 (12G5, PE) and anti-integrin-β7 (FIB504, PE), which were obtained from BD Bioscience. The anti-CD49d (HP2/1, FITC) antibody was obtained from Beckman Coulter (Fullerton, CA), and the anti-CD271 (LNGFR, PE and APC) antibodies were obtained from Miltenyi Biotec GmbH (Bergisch Gladbach, Germany).

### Measurement of hematological data

Two ml of blood was prepared every week. Blood samples were used to measure the white blood cell (WBC) count, red blood cell (RBC) count, hemoglobin (Hb) concentration, hematocrit values, mean corpuscular volume, mean cell hemoglobin concentration and platelet (PLT) count using a Sysmex K-4500 instrument (Toa-iyouddenshi, Kobe, Japan). The concentrations of the biochemical markers in blood samples were also monitored including total proteins, albumin, blood urea nitrogen, glucose, glutamic oxaloacetic transaminase, glutamic pyruvic transaminase, alkaline phosphatase, creatine phosphokinase, lactate dehydrogenase, creatine, sodium, potassium, chlorine and C-reactive protein using an AU400 instrument (Olympus Medical Systems, Tokyo, Japan).

### Quantification of gene-modified CD4+ T cells

The existence and persistence of genetically modified CD4+ T cells were monitored by measuring the proviral genome of the transgene using quantitative real-time PCR. DNA samples were extracted from 2×10^6^ PBMCs using a Gentra Puregene Blood Kit (QIAGEN, Hilden, Germany). The proviral copy number of the transgene was calculated from 400 ng of genomic DNA with quantitative PCR using a Cycleave RT-PCR Core Kit (Takara Bio) and Provirus Copy Number Detection Primer Set (Takara Bio) according to the manufacturer's instructions. The reaction was performed with the Thermal Cycler Dice Real Time System (Takara Bio), and the data was analyzed using Multiplate RQ software (Takara Bio). For each run, a standard curve was generated from the pMT-MFR-PL2 plasmid, whose copy numbers were already known. Based on the standard curve, the amount of infused cells was quantified.

### Detection of anti-MazF antibodies in macaque blood after transplantation of MazF-Tmac cells

To examine whether anti-MazF antibodies can be generated after the transplantation of MazF-Tmac cells, the plasma isolated from the macaques was analyzed. In order to detect anti-MazF antibodies, purified MazF protein or anti-monkey IgG (Nordic Immunological Laboratories, Tilburg, The Netherlands) was pre-coated onto the wells of a 96-well microplate and subsequently blocked with PBS-1% BSA. The plasma samples were isolated from the CD4T-2 at day 0, 33, 75, and 103 after transplantation and were diluted to 500,000-fold, 50,000-fold, and 10,000-fold. Cynomolgus macaque IgG purified from normal macaque plasma with Melon Gel IgG purification Kit (Thermo Fisher Scientific, Rockford, IL, USA) was used as a control for this reaction. The two-fold serial dilutions of the IgG (1 ng/ml to 64 ng/ml) and the diluted plasma samples, as described above, were separately added to each well. After an overnight incubation at 4°C, the wells were washed with PBS-1% BSA. The POD-conjugated anti-monkey IgG (Nordic Immunological Laboratories) was then added to the wells. After 4 hours of incubation at room temperature, the wells were washed three times with PBS-1% BSA followed by the addition of the substrate solution (o-Phenylenediamine, Sigma). The optical density of each well was read at 490/650 nm using a 680XR microplate reader (Bio-Rad Laboratories, Hercules, CA) after stopping the reaction with H_2_SO_4_ stop solution (Figure S1).

### Examination of the anti-viral efficacy of MazF-Tmac cells harvested from a monkey

To examine the function of the *mazF* gene in cells harvested from a MazF-Tmac-transplanted monkey, the frozen lymphoid cells from CD4T-1 at autopsy (214 days post-infusion of MazF-Tmac cells) were recovered, CD4+ T cells were selected using a CD4+ T Cell Isolation Kit (Miltenyi Biotec), stimulated with anti-CD3/CD28 beads at a cell-to-bead ration of 1∶1, and expanded in GT-T503 medium supplemented with 10% FBS, 200 IU recombinant human interleukin-2, 2 mM L-glutamine, 2.5 µg/ml Fungizone, 100 units/ml penicillin, and 100 µg/ml streptomycin. After 7 days of expansion, the genetically modified cells expressing ΔLNGFR+ were concentrated with an anti-CD271 monoclonal antibody (CD271 MicroBeads, Miltenyi Biotec) and expanded for 4 days. The cells from the CD271-negative fraction were also harvested and expanded as control non-gene modified CD4+ T cells. The expanded CD271-enriched cells and CD271-negative cells were infected with SHIV 89.6P at the MOI of 0.01 and cultured for 6 more days. Culture supernatants and cell pellets were harvested at 6 days post-infection. RNA in the culture supernatant was recovered with the QIAamp Viral RNA Mini Kit (QIAGEN) and SHIV RNA levels in the culture supernatant were determined by quantitative real-time PCR with a set of specific primers specific for the SHIV *gag* region [39]. In order to detect the Tat-dependent expression of MazF in the CD271-enriched MazF-Tmac cells harvested from the monkey, the cells were transduced with the Tat expression retroviral vector M-LTR-Tat-ZG [6] in the presence of RetroNectin® as per the manufacturer's instruction. Twenty hours after Tat transduction, the cells were harvested, counted by trypan blue exclusion assay, washed twice with PBS, and 5×10^5^ cells were suspended in 50 µl of 1× SDS sample buffer. The cell samples were incubated at 95°C for 10 min, and 5 µl of each cell sample was used for western blot analysis. For gel electrophoresis of proteins, the sample solutions described above were loaded into the wells of a 4–20% Tris-Glycine gel (Atto, Tokyo, Japan). After completion of electrophoresis, the gel was transferred to a polyvinylidene fluoride (PVDF) membrane (Millipore, Billerica, MA) with papers containing transfer buffer using the semi-dry method at 60 mA (constant voltage) for 60 min. The membrane was cut in half horizontally around the 20 kDa protein band of the pre-stained protein marker (Bio-Rad Laboratories). The upper part of the membrane was used to detect the α-tubulin (50 kDa) as an internal standard, while the lower part of the membrane was used to detect MazF (12 kDa). After blocking, the membranes were then incubated overnight at 4°C in the blocking buffer (5% skim milk in PBS) containing 1 µg/ml anti-α-tublin antibody (Cell Signaling Technology) and 1 µg/ml anti-MazF polyclonal antibody (rabbit, in-house preparation), respectively. Each membrane was washed three times and subsequently incubated at room temperature for 1 hour in 10 ml of the blocking buffer containing the 10,000-fold diluted goat anti-IgG rabbit antibody (peroxidase conjugated, Thermo Fisher Scientific). The membrane was washed five times by gentle shaking in the washing buffer at room temperature for 5 min. The membrane was soaked at room temperature for 5 min in substrate solution (SuperSignal West Femto Maximum Sensitivity Chemiluminescent Substrate, Thermo Scientific). Protein signals were detected by a CCD camera (LuminoShot 400 Jr, Takara Bio), which captures a digital image of the western blot.

### Collection of lymphocyte from several organs

Several organs were collected following euthanasia of the monkeys. After thoracotomy, the right atrium was incised, and 2 L of heparinized PBS was infused into the left ventricle using an 18-gauge needle. After perfusion, several organs were collected, and lymphocytes were separated using the following method: samples of spleen, thymus, liver, bone marrow, and axillary, inguinal and mesenteric LNs were minced and filtered through a 40 µm nylon filter (BD Bioscience); lymphocyte of the small intestine were collected by the Percoll (GE Healthcare, Castle Hill, Australia) density-gradient centrifugation method as described previously [39]; and lymphocytes obtained from each organ were used for the flow cytometric analysis, and extracted DNA was used for quantification PCR.

### *In vivo* homing analysis

CD4T-4 was used for homing analysis. Isolated CD4+ T cells were stimulated with anti-CD3/CD28 beads and cultured in GT-T503 medium supplemented with 10% FBS, 200 IU IL-2, 2 mM L-glutamine, and 2.5 µg/ml Fungizone. After 4 days of expansion, activated CD4+ T cells were divided into two culture bags (ClutiLife Eva), and 10 nM retinoic acid (Sigma) was added to one of the bags. After an additional 5 days of incubation, expanded cells with or without retinoic acid were harvested and labeled with 2 mM PKH26 (Sigma) or 5 µM CFSE (Sigma), respectively, according to the manufacturer's instructions. Thereafter, the cells were washed three times with PBS, mixed in PBS containing 10% autologous plasma and infused into the macaque. Then, CD4T-4 was euthanized at 3 days after transplantation. Lymphocytes from several organs were collected as previously described, and the distributions of labeled lymphocytes were detected by flow cytometric analysis. The specimens from several organs were fixed in buffered formaldehyde and embedded in plastic. Serial sections were made using a diamond saw. The slides were then analyzed under a fluorescence microscope to detect the distribution of the expanded cells in the distal organ specimens.

## Supporting Information

Figure S1 **Raw data of 490/650 nm absorbance.** The raw data of the optical density of each well at 490/650 nm was read using a microplate reader 680XR (Bio-Rad Laboratories, Hercules, CA) is represented.
(PDF)

Figure S2 **Photographs of histopathological analysis.** Individual photographic data of histopathological analysis of CD4T-1, -2, and -3 in Table 3 is represented.
(PDF)
