# Supplementary material for: In Vivo Safety and Persistence of Endoribonuclease Gene-Transduced CD4+ T Cells in Cynomolgus Macaques for HIV-1 Gene Therapy Model
Source: PLoS One. 2011 Aug 17;6(8):e23585. doi: 10.1371/journal.pone.0023585 (PMC3157387; doi:10.1371/journal.pone.0023585)
Supplement: Figure S1 — Raw data of 490/650 nm absorbance. The raw data of the optical density of each well at 490/650 nm was read using a microplate reader 680XR (Bio-Rad Laboratories, Hercules, CA) is represented. (PDF) [file pone.0023585.s001.pdf]

| IgG standard | |
|---|---|
| IgG (ng/mL) | Abs 490/650 nm |
| 64 | 1.197 |
| | 1.168 |
| 32 | 0.756 |
| | 0.764 |
| 16 | 0.439 |
| | 0.445 |
| 8 | 0.237 |
| | 0.232 |
| 4 | 0.12 |
| | 0.129 |
| 2 | 0.064 |
| | 0.069 |
| 1 | 0.051 |
| | 0.052 |

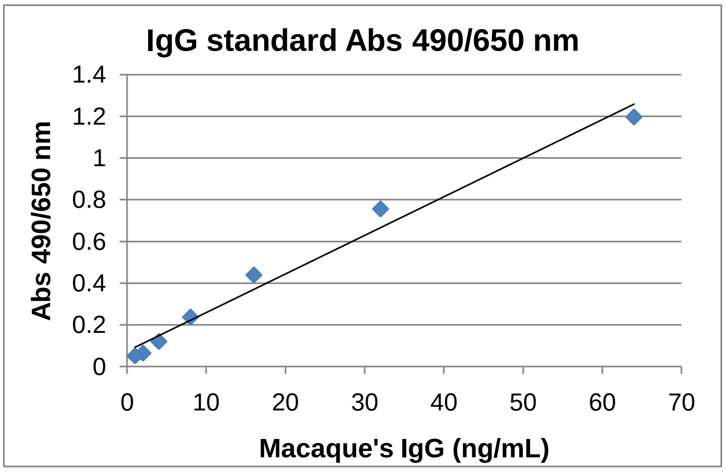


| anti-monkey IgG caoted plate | | |
|---|---|---|
| day after trasnplantation | Fold dilution of plasma | Abs 490/650 nm |
| 0 | 100000 | 0.994 |
| | | 0.999 |
| | 500000 | 0.290 |
| | | 0.290 |
| 33 | 100000 | 0.984 |
| | | 1.014 |
| | 500000 | 0.271 |
| | | 0.274 |
| 75 | 100000 | 0.996 |
| | | 1.041 |
| | 500000 | 0.288 |
| | | 0.294 |
| 89 | 100000 | 1.036 |
| | | 1.004 |
| | 500000 | 0.280 |
| | | 0.294 |
| 103 | 100000 | 1.129 |
| | | 1.156 |
| | 500000 | 0.341 |
| | | 0.346 |

| MazF coated plate | | |
|---|---|---|
| day after trasnplantation | Fold dilution of plasma | Abs 490/650 nm |
| 0 | 10000 | 0.022 |
| | | 0.006 |
| | 50000 | 0.006 |
| | | 0.010 |
| | 100000 | 0.006 |
| | | 0.010 |
| | 500000 | -0.001 |
| | | 0.001 |
| 33 | 10000 | 0.013 |
| | | 0.007 |
| | 50000 | 0.003 |
| | | 0.007 |
| | 100000 | -0.005 |
| | | -0.002 |
| | 500000 | -0.001 |
| | | -0.004 |
| 75 | 10000 | 0.012 |
| | | 0.021 |
| | 50000 | 0.009 |
| | | 0.005 |
| | 100000 | 0.003 |
| | | 0.003 |
| | 500000 | 0.000 |
| | | 0.001 |
| 89 | 10000 | 0.018 |
| | | 0.019 |
| | 50000 | 0.013 |
| | | 0.017 |
| | 100000 | 0.007 |
| | | 0.009 |
| | 500000 | 0.006 |
| | | 0.008 |
| 103 | 10000 | -0.005 |
| | | -0.001 |
| | 50000 | 0.007 |
| | | 0.004 |
| | 100000 | 0.005 |
| | | 0.002 |
| | 500000 | 0.007 |
| | | 0.003 |

Figure S1  Raw data of absorbance 490/650nm for Figure 3
