# Supplementary material for: In Vivo Safety and Persistence of Endoribonuclease Gene-Transduced CD4+ T Cells in Cynomolgus Macaques for HIV-1 Gene Therapy Model
Source: PLoS One. 2011 Aug 17;6(8):e23585. doi: 10.1371/journal.pone.0023585 (PMC3157387; doi:10.1371/journal.pone.0023585)
Supplement: Figure S2 — Photographs of histopathological analysis. Individual photographic data of histopathological analysis of CD4T-1, -2, and -3 in Table 3 is represented. (PDF) [file pone.0023585.s002.pdf]

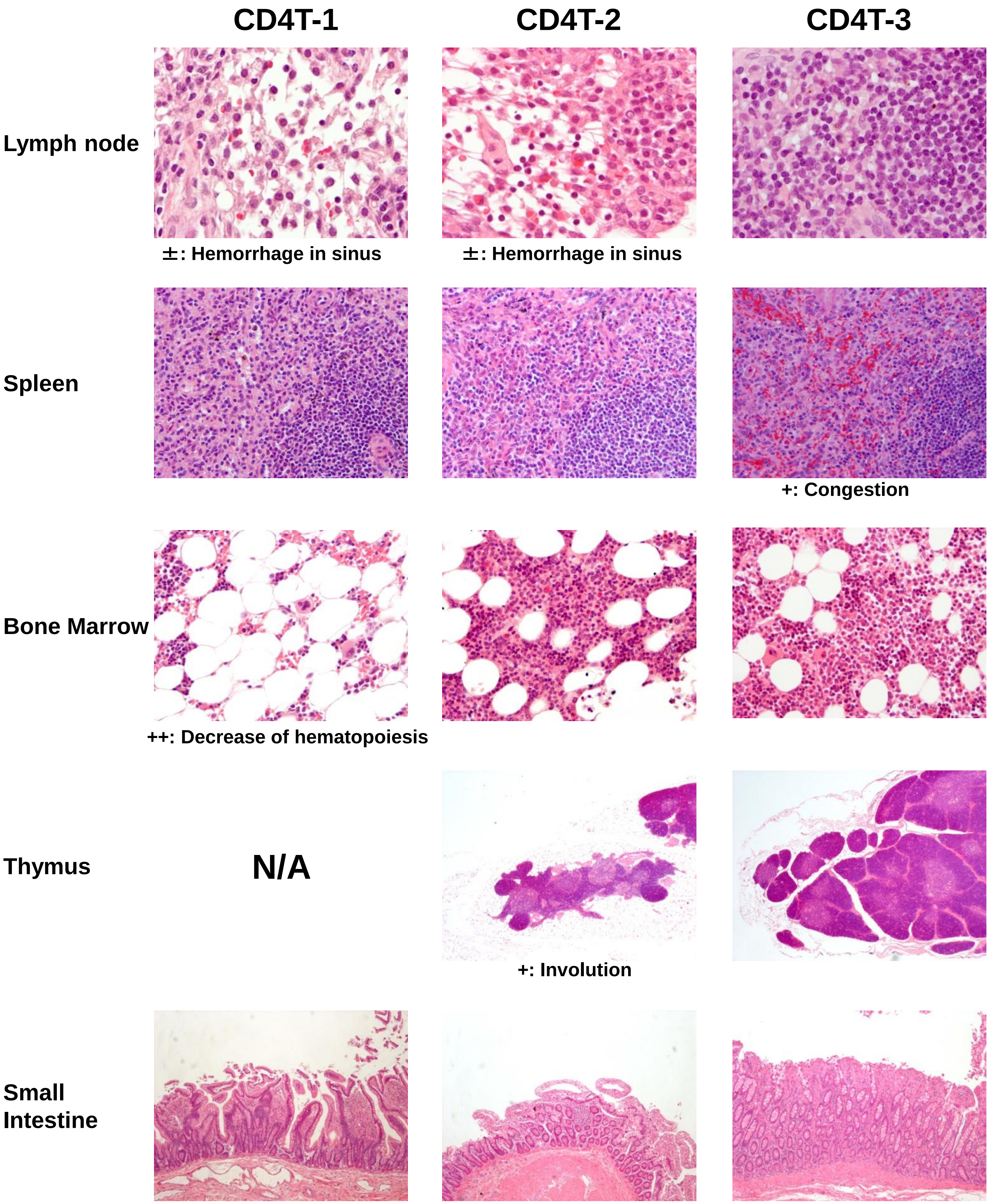

Figure S2 Photographs of histopathological analysis.

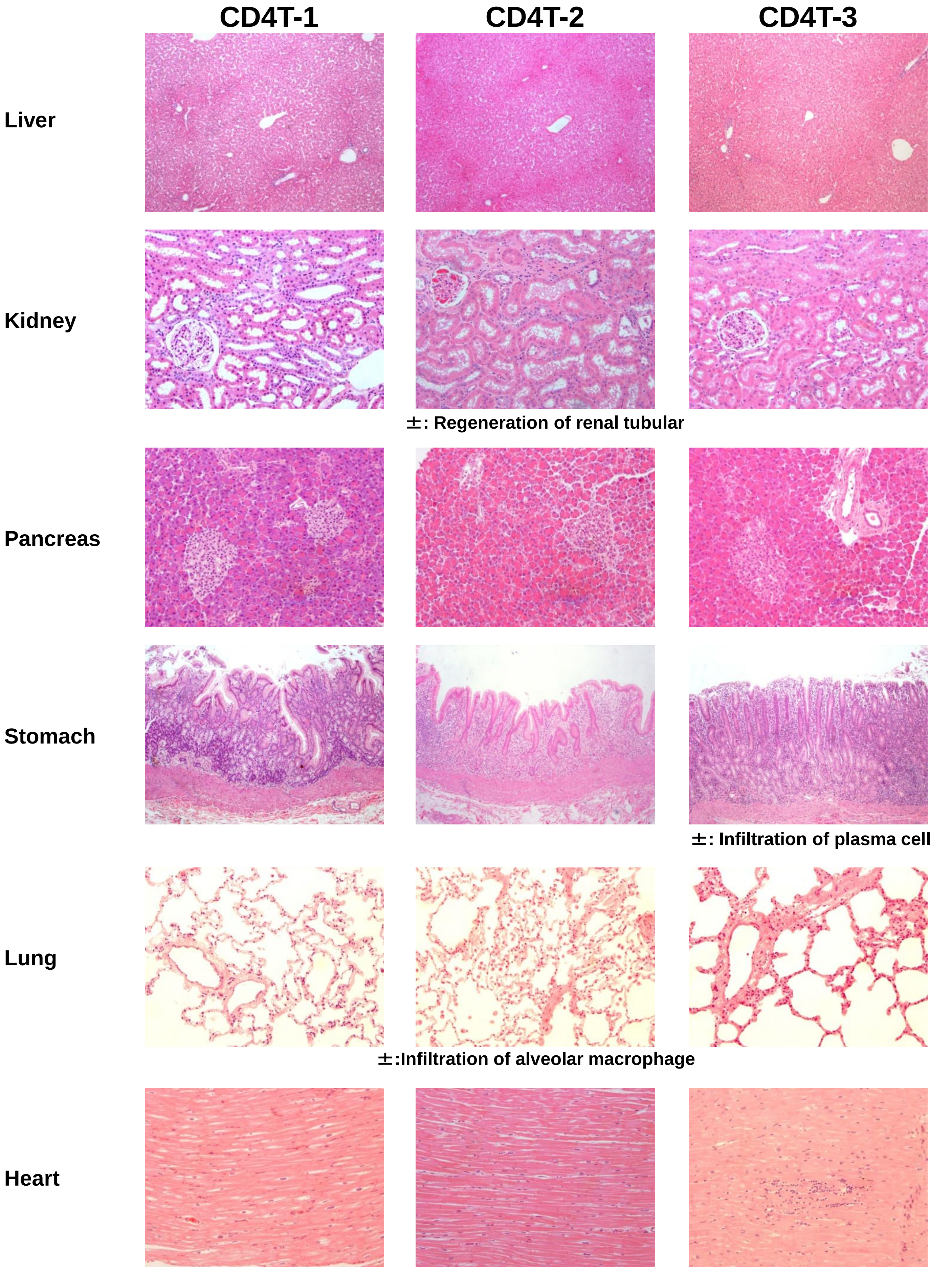

Figure S2 Photographs of histopathological analysis.
